# Advancements in delivery strategies and non-tissue culture regeneration systems for plant genetic transformation

**DOI:** 10.1007/s44307-024-00041-9

**Published:** 2024-09-26

**Authors:** Minyi Wu, Ao Chen, Xiaomeng Li, Xiaoyun Li, Xingliang Hou, Xu Liu

**Affiliations:** 1 Guangdong Provincial Key Laboratory of Applied Botany, South China, Botanical Garden, Chinese Academy of Sciences, Guangzhou, China; 2 University of the Chinese Academy of Sciences, Beijing, China; 3 Guangdong Provincial Key Laboratory of Biotechnology for Plant Development, School of Life Science, South China Normal University, Guangzhou, China

**Keywords:** Plant genetic transformation, Delivery strategy, Non-tissue culture, Plant regeneration system, *In planta* transformation

## Abstract

Plant genetic transformation is a pivotal and essential step in modifying important agronomic traits using biotechnological tools, which primarily depend on the efficacy of transgene delivery and the plant regeneration system. Over the years, advancements in the development of delivery methods and regeneration systems have contributed to plant engineering and molecular breeding. Recent studies have demonstrated that the efficiency of plant transformation can be improved by simultaneously delivering meristem-developmental regulators, utilizing virus-mediated gene editing, and executing non-sterile *in planta* manipulations. Efficient genetic delivery and non-tissue culture regeneration systems are gradually being developed. This review summarizes diverse delivery strategies and *in planta* regeneration techniques aimed at improving the efficiency of plant genetic transformation. We also emphasize the integration and utilization of these emerging transgenic approaches for expediting future crop engineering.

## Introduction

Plant genetic transformation is a crucial technological advance in modern scientific application, contributing to the understanding of plant science and opening a new era of crop engineering (Perl et al. 1993; Su et al. 2023). The accuracy of genetic modification depends on the target genetic transformation in plants; however, the genetic transformation of different crops varies, and many plants cannot be transformed or the process is inefficient or laborious. Many studies have shown that target DNA can be transferred and integrated into the plant genome through diverse genetic delivery strategies and that stable transgenic plants can be propagated via an efficient regeneration system (Su et al. 2023). The efficiency of genetic transformation predominantly depends on the ability to deliver transgenes and subsequent germinal regeneration (Ramkumar et al. 2020). Thus, plant genetic transformation can be improved by achieving transgene delivery and optimizing regeneration systems, and studying gene delivery techniques and regeneration systems in plants is crucial for advancing crop breeding. However, significant knowledge gaps remain in these processes that require our attention.

Exogenous DNA is transferred into the plant genome or intracellular target(s) through various delivery strategies, including particle bombardment, nanoparticle infection, viral mediation, and *Agrobacterium*-mediated techniques. Particle bombardment-mediated transformation is conducted on cells, calluses, or organs irrespective of the location of the plant tissue (Bonawitz et al. 2019). Nanoparticle-mediated transformation enables the acquisition of stable genetic material in specific species (Zhao et al. 2017) and allows for genetic transformation regardless of genotype (Wang et al. 2022b). Virus particles are an effective tool for delivering foreign DNA and facilitating rapid transformation, and virus-mediated plant gene editing technology can be used to generate transgenic plants without tissue culture (Liu et al. 2023b). Compared with these particle-mediated direct transformations, the classical *Agrobacterium*-mediated indirect transformation is not restricted by plant tissues and exhibits broad applicability with high efficiency and stability (Tzfira and Citovsky 2006). Furthermore, in the pursuit of enhancing plant transformation, several developmental regulators used to regenerate adventitious buds have been identified through the ectopic expression of *Baby boom* (*BBM*), *Wuschel 2* (*WUS2*), or *Growth-regulating factor* (*GRF*) (Wang et al. 2020; Chen et al. 2022a; b).

In addition to a delivery system, an efficient plant regeneration system is necessary for plant genetic transformation. Existing plant regeneration methods rely mainly on tissue culture techniques. However, tissue culture requires aseptic operation, which is laborious and costly, and usually reduces the efficiency of plant genetic transformation (Cao et al. 2022; Mei et al. 2024). In recent years, researchers have shown increasing interest in establishing plant regeneration systems via non-sterile manipulation techniques. Compared with traditional plant regeneration methods, different genetic delivery strategies combined with innovative *in planta* regeneration systems have the potential advantages of being simple and tissue culture-independent. Here, we review diverse delivery and regeneration systems for plant genetic transformation and highlight their potential applications in the future of crop breeding.

## Delivery methods for plant genetic transformation

### Particle bombardment delivery

Particle bombardment is a commonly used plant genetic transformation technology. Through high-speed particle bombardment, the desired foreign plasmid is directly introduced into plant cells, and the carried genetic fragments are transferred and integrated into the plant genome (Sanford 1990). Compared with other transformation methods, particle bombardment can directly transform various plant tissues and species, regardless of the genetic background of the species. Particle bombardment is suitable for the transient transformation of plants and stable inheritance through tissue culture. In 1988, Klein and colleagues introduced foreign genes into corn (*Zea mays*) tissue culture cells via particle bombardment without removing the cell wall, successfully delivering DNA to embryonic tissues (Klein et al. 1988a). Simultaneously, this research team achieved stable transformation of tobacco by bombarding intact cells and tissues with DNA-coated particles (Klein et al. 1988b). Subsequently, many studies have demonstrated the suitability of this method for different crops, such as soybean (*Glycine max*), barley (*Hordeum vulgare*), cabbage (*Brassica rapa*), tomato (*Solanum lycopersicum*), poplar (*Populus tremula*), and wheat (*Triticum aestivum*) (Horsch et al. 1985; Christou et al. 1988; Mendel et al. 1989; Vasil et al. 1993; Liu et al. 2024b; Tanwar et al. 2024). The method relies on a particle bombardment device (also known as a ‘gene gun’) to introduce foreign DNA into plant cells. The gene gun contains a gas pressurization device that has developed from low-efficiency gunpowder versions to pneumatic and discharge types and is efficiently applied to various plant organs and tissues. The advantages of the pneumatic gene gun are controllable parameters, high success rates, and reasonable cost, with uses including the realization of transgene events in poplar trees and the medicinal plant safflower (Ye et al. 2014; Xian et al. 2023). Recently, a gene gun-mediated instantaneous expression system of the GRF-GIF1 (GRF interacting factor 1) complex was established, which improved the regenerative ability of wheat and achieved efficient genome editing (Qiu et al. 2022). Applying the gene gun method to different crops requires adjustment of the bombardment parameters, such as the number of bombardments, pressure, and distance, as these factors can affect the final transformation efficiency.

### Nanoparticle-mediated delivery

Nanoparticle-mediated plant genetic transformation was first realized by delivering foreign plasmids into tobacco suspension cells via silicon carbide fibers (Kaeppler et al. 1992). In plant cells, foreign T-DNA from nanoparticles is recognized and integrated into the genome for stable genetic transformation. Nanoparticles have substantial potential as biomolecular delivery materials owing to their cell-penetrating ability, wide applicability, and use in precisely targeting plant cells to regulate gene expression and tissue distribution (Ramasamy et al. 2023). In the early stages of this method, external forces were needed to deliver nanoparticle-mediated plasmids or proteins into plant cells (Torney et al. 2007). Subsequently, a functionalized mesoporous silica nanoparticle was used to construct a transient system and deliver foreign DNA into *Arabidopsis* root without mechanical force (Chang et al. 2013). This method can efficiently deliver DNA for the transient high expression of RNA or protein in leaves or protoplasts from tobacco (*Nicotiana tabacum*), Arugula (*Eruca sativa*), wheat, spinach (*Spinacia oleracea*), and cotton (*Gossypium hirsutum*), markedly contributing to the study of gene function in these plants (Demirer et al. 2019; Kwak et al. 2019). Stable transformation using this approach was first achieved with the transfer of magnetic nanoparticles with foreign DNA to pollen via a magnetic field and screening for stable transgenic seeds and seedlings (Zhao et al. 2017). A novel maize transformation system independent of genotype was developed using nanomagnetic beads under optimal conditions for pollen viability and pore opening, overcoming the challenges of tissue culture and genotype restrictions in maize genetic transformation (Wang et al. 2020). However, nanoparticle-mediated genetic transformation usually has low efficiency, and even if the nanoparticles can penetrate the plant cells, the success rate of transformation is limited. In addition, the stabilization of nanoparticles in plants is a challenge, with nanoparticles aggregating, precipitating, or decomposing, and this changeable environment reduces the stability of genetic transformation. Continuous research and technical improvements are necessary to overcome the challenges and shortcomings in nanoparticle-mediated transformation. An example of one such improvement is polyvinylimide-modified single-wall carbon nanotubes, which have been shown to adsorb nucleic acids electrostatically and protect them from nuclease degradation. (Demirer et al. 2019). In addition, pollen magnetic transfection has been found to substantially improve the efficiency of genetic transformation, eliminate species dependence, and achieve high-throughput screening and multigene co-transformation (Yan et al. 2022). They have the potential to accelerate the speed of molecular breeding, and in the future, nanoparticle-mediated delivery systems can be developed with reference to these methods.

### Virus vector-mediated delivery

Virus-mediated genetic transformation is a common method of plant gene transformation in which a virus functions as a carrier to infect target host cells and tissues and introduce foreign DNA or RNA fragments (Rössner et al. 2022). The vectors used for plant viral transformation mainly comprise double-stranded DNA virus vectors (e.g., cauliflower mosaic virus) and single-stranded RNA virus vectors (e.g., tobacco mosaic virus) (Baltes et al. 2014; Cody et al. 2017). Recently, engineered plant RNA virus vectors were used to construct a gene editing system, and these approaches effectively produced genetically targeted gene editing in plants, marking the beginning of virus-induced gene editing (VIGE) for plants (Ellison et al. 2020; Ma et al. 2020; Li et al. 2021). In addition, a technical problem in studying negative-strand plant RNA viruses was solved by using rhabdovirus as a model and achieving infectious cloning of the full-length cDNA of the virus and generating a recombinant virus with similar infectious activity as the wild-type virus (Wang et al. 2022a). Liu and colleagues subsequently established a transient delivery system based on a wide-host tomato spotted wilt virus (TSWV), providing a promising solution for genome editing in numerous crops (Liu et al. 2023a). Owing to the small size of inserted foreign DNA fragments, delivering a valuable gene with large fragments is difficult using the virus vector approach. However, Yin et al. constructed a chimeric modification system for pepper mild mottle virus via Cre/loxP recombination, providing a new method for constructing large-genome viruses and modifying chimeric viruses (Yin et al. 2022). Despite the reported successes, vector-mediated genetic transformation still has some shortcomings, including that the host plant is prone to disease due to virus infection. In addition, the method is more suitable for transient transformation because of the difficulty in achieving stable inheritance of foreign genes in offspring.

### *Agrobacterium*-mediated delivery

*Agrobacterium*-mediated genetic transformation involves embedding foreign genes into the binary plasmid of *Agrobacterium*, then transferring and integrating the foreign T-DNA into the plant genome by infecting recipient plants to achieve instantaneous or stable transformants. In the early stages of the development of this method, Smith and Townsend reported that *Agrobacterium tumefaciens* was the pathogen of crown gall and could infect plants widely (Smith and Townsend 1907). In the process of infecting plants, *Agrobacterium tumefaciens* was found to transfer T-DNA and viral proteins from bacteria to host plant cells (Chilton et al. 1977). In 1985, tobacco transformation was effectively induced by using leaf disc tissue co-cultured with *Agrobacterium* (Horsch et al. 1985). Notably, the T-DNA was transferred to the developing embryo and integrated into its genome by immersing the developing flower of *Arabidopsis thaliana* in an improved *Agrobacterium* solution (Clough and Bent 1998). Until recently, *Agrobacterium*-mediated genetic transformation, which is the oldest plant transgenic method, was the most prevalent plant transgenic method. To improve the transformation efficiency of the *Agrobacterium* system, many adaptations have been applied to different plants. For example, stem cell-regulated genes (*MdWUS*, *MdBBM*, or *MdGRF5*) were used in fruit tree transformation with *Agrobacterium rhizogenes* to integrate foreign genes into hairy roots and differentiate them into buds, ultimately producing complete transgenic fruits via hairy root regeneration systems (Liu et al. 2024a). Similarly, several crops, such as watermelon (*Citrullus lanatus*), apricot (*Armeniaca vulgaris*), and pigeon pea (*Cajanus cajan*), produce transgenic calli or sprouts via the *Agrobacterium rhizogenes*-mediated method (Zhao et al. 2024; Jedličková et al. 2024; Meng et al. 2019). Moreover, *Agrobacterium tumefaciens* was transformed into pears and peanuts via leaf-cutting methods or the pollen tube pathway, improving their regeneration and transformation efficiency (Zhai et al. 2022; Xue et al. 2023; Zhou et al. 2022). *Agrobacterium tumefaciens*-mediated transformation is easily restricted by distinct explants or genotypes. Therefore, developing methods that do not require tissue culture and can overcome genotypic constraints is crucial. Studies have shown that plants with strong regenerative ability can generate stable transgenic plants quickly and efficiently without tissue culture or genotype restriction (Cao et al. 2022; Mei et al. 2024). For some crops with weak regenerative capacity, delivery efficiency is a major consideration for the future.

### Electroporation and PEG-mediated delivery methods

Polyethylene glycol (PEG)-mediated transformation is the process of inducing plant cells or protoplasts to take up foreign DNA under conditions that include PEG, calcium chloride, and elevated pH. Krens and colleagues were the first to use PEG for the genetic transformation of tobacco protoplasts (Krens et al. 1982). PEG-mediated protoplast transformation is currently the most widely used transient expression system in *Arabidopsis thaliana* mesophyll protoplasts. After removal of the cell wall by enzymatic digestion, DNA is delivered into the protoplasts by PEG- and calcium-mediated transformation, which is rapid, does not require aseptic manipulation, and allows the transformation of co-expressed genes (Yoo et al. 2007). In addition, the electroporation method uses high-voltage electrical pulses on the protoplast membrane to stimulate electrical perforation, thereby promoting the uptake of foreign DNA (Shigekawa and Dower 1988). PEG-mediated transformation and electroporation are the main methods used for transient expression in protoplasts, but further application of these techniques is hindered by their low efficiency and difficulty in protoplast regeneration. A similar method is the vacuum infiltration transformation method, which involves transferring a suspension containing *Agrobacterium* cells into plants through vacuum pressure (Bechtold et al. 1998). This vacuum method is quick and easy but essentially belongs to *Agrobacterium*-mediated genetic transformation, and its feasibility is low in most plants.

### Transgene-free gene editing delivery system

The gene editing method accurately modifies specific target sequences of the organismal genome by inducing DNA repair and mutation (Chen et al. 2019; Gao 2021; Li et al. 2024). The clustered regularly interspaced short palindromic repeats (CRISPR)/CRISPR-associated protein 9 (CRISPR/Cas9) system is the most widely used technology for gene editing; however, the function of this system requires the introduction and integration of exogenous elements carrying the CRISPR/Cas9 cassette and transgenic screening markers into the genome of recipient plants, and residual non-integrated components may also be transmitted in plant cells, causing adverse effects. Similar problems are encountered with other common transformation techniques, therefore it is worth considering how to remove transgene elements from the genome after finishing gene editing. In recent years, several trials have been conducted with the aim of resolving this issue. For example, adding an RNA interference (RNAi) element to the CRISPR/Cas9 structure resulted in transgenic rice that was sensitive to bentazon, allowing only transgene-free seedlings to grow normally (Lu et al. 2017). The transgene killer CRISPR (TKC) system was exploited to achieve self-elimination after gene editing because the suicide element can specifically kill pollen and seeds containing the transgene to autonomously screen transgene-free and edited plants (He et al. 2018). Owing to the extreme dependence of somatic embryo culture and regeneration on genotypes in cotton, regenerated plants can be obtained from only a few genotypes; thus, an efficient transformation system was established for cotton that overcomes the restriction of genotype (Ge et al. 2023). These studies provide valuable reference models for transgene-free plant transformation by removing transgene markers. Although self-crossing crops can eliminate transgene traces via self-breeding, self-incompatible crops cannot accomplish self-crossing. A potential solution to this problem was developed by using inducible site-specific recombinase and excising activated markers; however, the addition of recombinase appeared to affect the viability of some tissues (Yin et al. 2022). Fortunately, the CRISPR/Cas9 element was inserted into an engineered plant negative-stranded RNA virus vector and DNA virus; thus, the viral genome cannot be integrated into the host chromosome, but the CRISPR/Cas9 elements are expressed and function in the recipient genome (Ma et al. 2020; Vu et al. 2020; Zhang et al. 2024). For non-sterile systems, further research is needed to determine how traces of transgenes can be removed during plant transformation, especially for plants with self-incompatibility.

### Regeneration systems for *in planta* genetic transformation

Transformed clones are regenerated predominantly via aseptic tissue culture techniques; however, there is some evidence that independently transformed plants can be regenerated under non-sterile *in planta* conditions. In addition, specific regeneration factors can activate the *in planta* regeneration of adventitious shoots and improve the efficiency of plant genetic transformation by altering the regeneration mode of plants.

### Developmental regulator-mediated regeneration systems

In monocotyledonous and dicotyledonous plants, genes encoding meristem-developmental regulators effectively increase plant regeneration efficiency (Kong et al. 2020). The ectopic expression of an AP2/ERF transcription factor did not cause injury but regenerated adventitious buds from root explants on the regeneration medium without auxin pretreatment (Iwase et al. 2015). In addition, co-transformation of *BBM* and *WUS2* promoted transformant regeneration in immature sorghum, sugarcane, and rice callus embryos (Lowe et al. 2016). Similarly, the transformation efficiency of transgenic apples was significantly improved by ectopic expression of the *BBM* gene (Chen et al. 2022a). However, ectopic *BBM*/*WUS2* expression can also reduce the quality of regenerated plants and lead to sterility (Wang et al. 2020). In addition to *BBM*/*WUS2* co-transformation, GRF4-GIF1-BBM co-transformation markedly improved the genetic transformation efficiency of maize without tissue culture callus induction (Chen et al. 2022b). Moreover, wheat GRF4-GIF1 fusion protein could improve regeneration efficiency in wheat, triticale, rice, and citrus to obtain fertile genetic progeny (Debernardi et al. 2020). Similarly, ectopic expression of *AtGRF5* and homologous genes in callus cells could promote bud formation, significantly improving transformation efficiency, and fertile transgenic plants were obtained from sugar beet, corn, and other crops (Kong et al. 2020).

As research continues, genotypic dependence remains a key factor limiting plant genetic transformation. Overexpression of *TaWOX5* could significantly improve the transformation efficiency of wheat, rye, maize, and barley, while *TaWOX5* expression in various wheat calli did not inhibit stem differentiation or root development, indicating that the degree of genotypic dependence of this method is less than that of other methods (Wang et al. 2022a). Similarly, the transcription factor TaDOF (DNA binding with one finger) could improve the callus induction rate and transformation efficiency in wheat (Liu et al. 2023b). Furthermore, genes encoding the meristem determination regulators *Plethora 5* (*PL5*), *WUS*, and *BBM* were directly inserted into segments of a binary vector to induce the developmental fate of specific transformed cells and facilitate their differentiation into mature tissues (Lian et al. 2022). However, since calli must be induced via tissue culture to produce transgenic plants, the above methods are time consuming and experience contamination issues, and ectopic expression of the developmental regulators may have adverse effects on plant growth, development, and viability, potentially affecting final transformation efficiency (Gordon et al. 2019).

Two novel genetic modification methods were established in tobacco via *Agrobacterium* carrying developmental regulators without callus induction (Cody et al. 2023). One is the fast-treated *Agrobacterium* co-culture (Fast-TrACC) method, which delivers developmental regulators to germ-free growing seedlings, causing the meristem to produce regenerated buds that eventually form complete regenerated plants. The second method is direct delivery, where developmental regulators are directly introduced into plants whose meristem has been completely removed (Cody et al. 2023). Developmental regulators can promote the differentiation of callus cells and the formation of adventitious shoots without external stimuli, therefore genetic transformation can be achieved during the process of regeneration. This means that the transformation process can be achieved by inducing new meristems through the delivery of developmental regulators and transferring target genes to the meristem, ensuring stable, heritable transgenic plants.

Recent years have seen the rapid development of multiomics in plant science. In barley, developmental regulators that promote embryogenic callus formation have been identified via transcriptomics, and their discovery holds considerable potential for efficient genetic transformation (Suo et al. 2021). In the future, combining omics techniques may facilitate the identification of more developmental regulators and specific expression regulatory elements, which should enhance the regeneration ability of plants and transformation efficiency.

### Plant non-tissue culture regeneration systems

In traditional plant transformation, the transformed callus and stem cells propagate primarily through tissue culture, with plant tissue culture being a sophisticated technology for the specific growth of plant cells, tissues, or organs under sterile conditions (Haque et al. 2022). Tissue culture is used in many fields, including basic research and plant breeding, and most DNA delivery methods predominantly rely on tissue culture regeneration systems. However, tissue culture is limited by genotype, is operationally expensive, and requires a sterile environment, all of which hinder the application and exploration of this technique. Therefore, developing simple and optimized regeneration systems for efficient delivery is vital for plant genetic transformation.

Recently, researchers have explored approaches for efficiently delivering genes into plants under non-sterile conditions. In 2020, Maher and colleagues used aseptically cultured tobacco seedlings combined with developmental regulators and gene editing technology and infected the incisions of tobacco seedlings with *Agrobacterium* via the Fast-TrACC method, which induced the production of meristem-like structures in the plants without the need for tissue culture, thus facilitating successful gene editing (Maher et al. 2020). In the same year, a highly efficient genotype-independent strategy was established in which an RNA virus vector was used to achieve gene editing in tobacco without tissue culture (Ellison et al. 2020). The following year, Li and colleagues reported the use of a modified RNA virus (barley stripe mosaic virus) vector to express single guide RNA and effectively edit genes in Cas9 transgenic wheat plants via non-tissue culture (Li et al. 2021). In rice, a technique was developed to achieve genetic transformation without tissue culture by detecting Cas9 protein in the genome of the recipient variety, Malaysian rice M219, whose embryonic meristem acts as the target tissue for *Agrobacterium* penetration (Tamizi et al. 2023). In 2022, the cut–dip–budding (CDB) delivery method was proposed, which uses an *Agrobacterium rhizogenes*-mediated regeneration system to deliver CRISPR/Cas9 elements into plants, achieving GFP expression and *PDS* gene editing in various plants, including rubber roots (*Taraxacum kok-saghyz*), sweet potatoes (*Ipomoea batatas*), and toon trees (*Ailanthus altissima*) (Cao et al. 2022). In this method, the plants are cut, then inoculated with *Agrobacterium rhizogenes* at the cutting sites to induce hairy roots, and finally, genetically stable transgenic plants are obtained via root suckering. The CDB method is easy to perform and does not require an aseptic operation process. The same research team subsequently used the method to obtain transgenic medicinal plants and succulent plants, including pugongying (*Taraxacum mongolicum*), yuanzhi (*Polygala tenuifolia*), and dihuang (*Rehmannia glutinosa*) (Cao et al. 2024; Lu et al. 2024). The applications of the CDB method rely on *Agrobacterium rhizogenes*, which potentially causes abnormal plant growth owing to the random insertion of exogenous *rol* genes (Tepfer 1984; Dehio et al. 1993; Kumlehn et al. 2006).

Simple non-sterile transformation methods with strong regenerative ability have been reported in sweet potato (Liu et al. 2022, Zhang et al. 2023). Subsequently, Mei et al. developed a simple and quick *in planta* transformation system named Regenerative Activity-dependent *in Planta* Injection Delivery (RAPID), which uses unarmed *Agrobacterium tumefaciens* to deliver target DNA into plant meristematic cells and then regenerates independent transgenic plants via vegetative propagation (Mei et al. 2024). Researchers previously used soaking, vacuum, and injection operations to test transformation in sweet potato, but only the injection method for stem segments could quickly yield positive transformants. The detached stem segments were placed in soil for cutting culture, and transgenic leaves, shoots, and tubers could be quickly obtained for different needs. Applying this RAPID system approach, potato (*Solanum tuberosum*) and bayhops (*Ipomoea pes-caprae*) were efficiently transformed (Mei et al. 2024). Compared with traditional transformation methods, RAPID has greater transformation efficiency, is quicker, and does not require complex tissue culture procedures. In addition, RAPID is a potential transformation tool for a wide range of plant species with high regenerative capacity. Recently, a novel virus-induced gene silencing (VIGS) system was developed in sweet potato via *Agrobacterium tumefaciens* and the RAPID approach, which successfully established a rapid and simple virus-induced transformation system (Zhang et al. 2024). Collectively, these non-sterile methods are simple, efficient, universal, and genotype-independent and offer insights into the integrated utilization of delivery and regeneration systems for plant transformation.

## Summary and perspectives

**Fig. 1:**
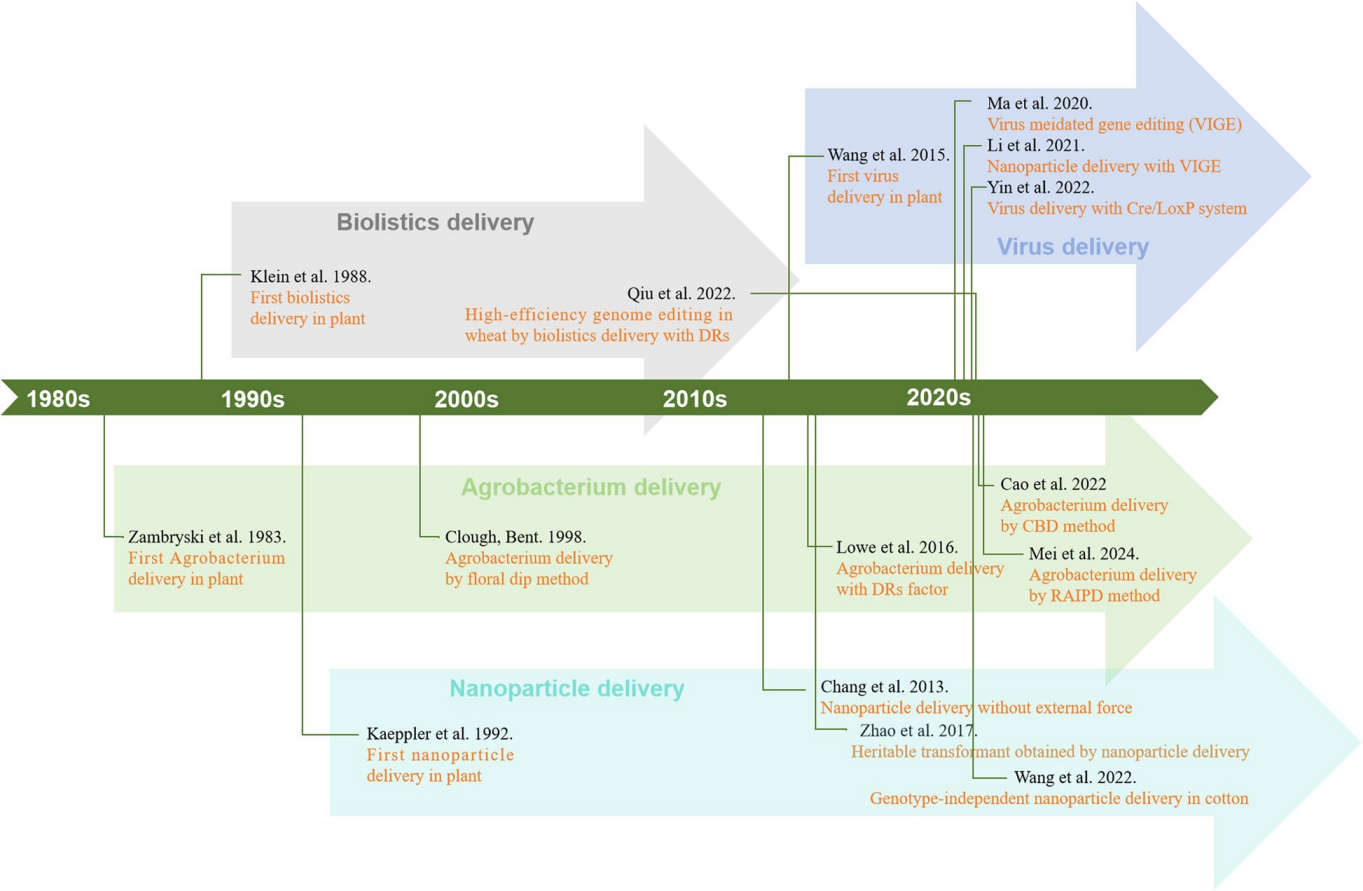
Timeline of delivery systems for plant genetic transformation. Note: DRs, developmental regulators; CBD, cut–dip–budding delivery method; RAIPD, Regenerative Activity-dependent *in Planta* Injection Delivery

**Fig. 2:**
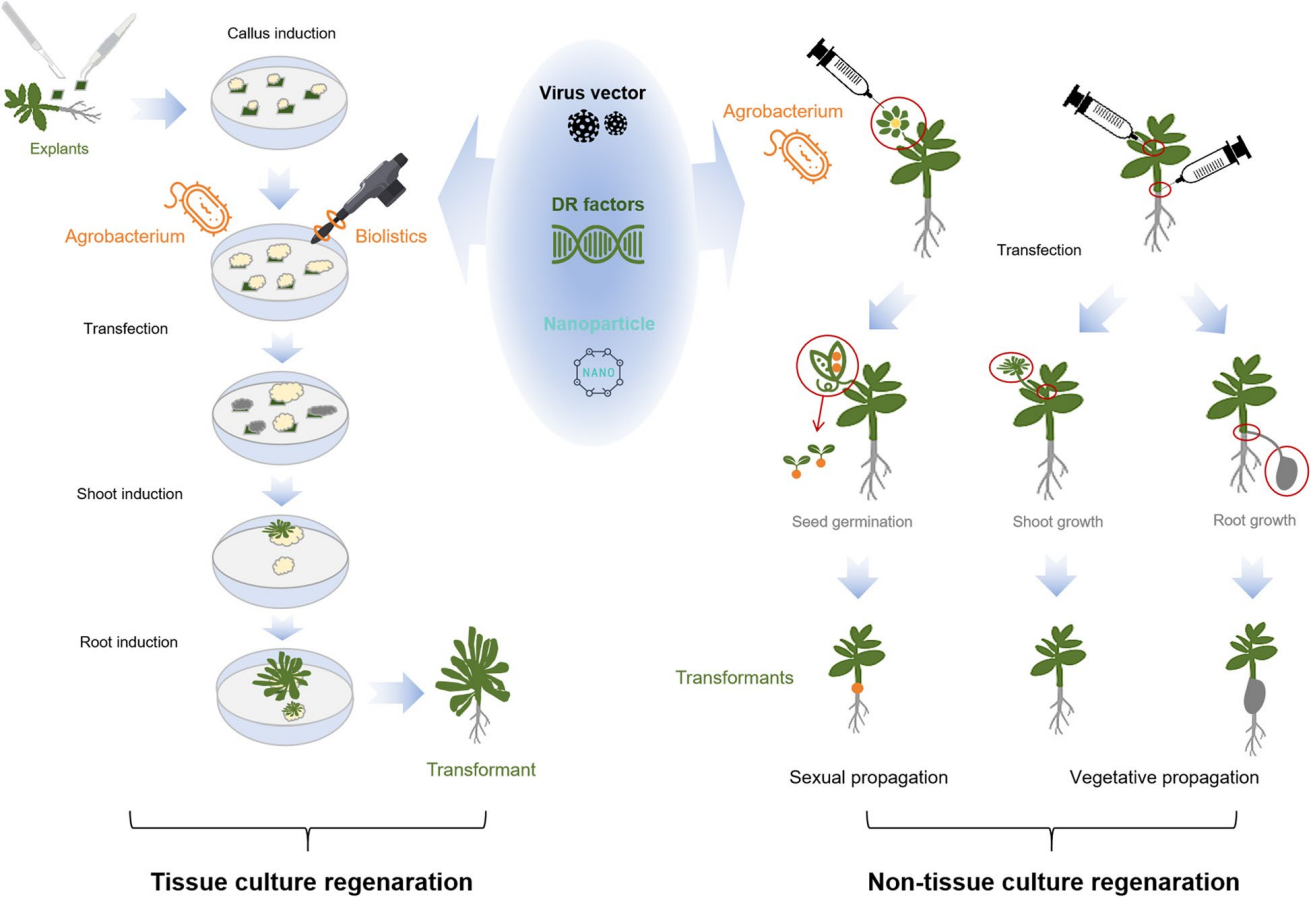
Tissue culture and non-tissue culture regeneration systems for plant transformation

Improving delivery and regeneration efficiency is crucial in plant genetic transformation systems. In this review, we summarized the prevalent delivery and regeneration techniques used for different transformation systems in various plants (Table 1; Fig. 1). Devising a universally applicable and highly efficient genetic transformation technique for diverse plants or crop species is imperative. Traditional methods of gene delivery and regeneration through tissue culture are delicate and time-consuming; consequently, the recently reported *in planta* delivery and regeneration methods without tissue culture can markedly accelerate the process of plant transformation (Table 2; Fig. 2). However, these non-sterile methods are only suitable for plants with strong regeneration capabilities, such as sweet potato, and some reported methods may lead to transgenic plants with excess genome insertions from *Agrobacterium rhizogenes* or developmental regulators. Thus, the methods might not be applicable for most plants with poor regeneration capabilities (Cao et al. 2022; Mei et al. 2024). Plant developmental regulators reliably improve regeneration efficiency for plant transformation, and multiomics techniques should facilitate the discovery of developmental regulators and other regulatory elements that will improve the efficiency and compatibility of plant regeneration and delivery systems. However, ectopic expression of some developmental regulators, such as BBM/WUS2, may have negative effects on plant growth and development and may affect reproduction. Specific promoters or recombinases have been reported to potentially remove exogenous genes (Lowe et al. 2018; Wang et al. 2020), but the low efficiency of these techniques may not completely address the problems. Therefore, developing next-generation techniques that improve compatibility between the effects of additional factors and transgene-free manipulation is important and should revolutionize plant transformation processes. Current non-sterile systems only target a limited number of plants, and understanding how to effectively remove transgene traces from plant transformation is unclear and warrants further investigation, especially for plants with self-incompatibility. In conclusion, elucidating how to use developmental regulator-mediated regeneration, transgene-free operation, and non-sterile *in planta* systems to facilitate plant genetic transformation will significantly advance modern plant engineering and subsequently further the study of plant genetics and crop molecular breeding.

**Table 1:** List of the representative systems for plant genetic transformation

Plant species	Delivery system	Tissue culture	Types of hereditabilit**y**	DR factors	Reference**s**
Sweet potato, *Taraxacum kok-saghyz*, *Coronilla varia*, *Ailanthus altissima*, *Aralia elata*, and *Clerodendrum chinense*	ARMT	×	SI	×	Cao et al. 2022
*Taraxacum mongolicum*, and *Rehmannia glutinosa*	ARMT	×	SI	×	Cao et al. 2024
Apple	ARMT	○	SI	○	Liu et al. 2024a
*Kalanche blossfeldiana*, *Crassula arborescens* and *Sansevieria trifasciata*	ARMT	×	SI	×	Lu et al. 2024
*Taraxacum platycarpum*	ARMT	○	SI	×	Lee et al. 2004
Tobacco	ATMT	○	SI	×	Shoyeb et al. 2020
*Hevea brasiliensis*	ATMT	○	SI	×	Dai et al. 2021
Sweet potato	ATMT	○	SI	×	Choi et al. 2007
Maize	ATMT	○	SI	○	Chen et al. 2022b
Wheat	ATMT	○	SI	○	Debernardi et al. 2020
Cotton	ATMT	○	SI	×	Ge et al. 2023
Soybean, Canola, Sunflower, and Maize	ATMT	○	SI	○	Kong et al. 2020
Wheat	ATMT	×	SI	×	Li et al. 2021
Sorghum, Sugarcane, and Rice	ATMT	○	SI	○	Lowe et al. 2016
Sweet potato, Potato, and *Ipomoea pes-caprae*	ATMT	×	SI	×	Mei et al. 2024
Citrus	ATMT	○	SI	×	Ramasamy et al. 2023
Wheat	ATMT	○	SI	○	Wang et al. 2022a
Soybean	ATMT	-	TT	×	Wang et al. 2021
Safflower	ATMT	-	TT	×	Xian et al. 2023
Pear	ATMT	○	SI	×	Xue et al. 2023
Peanut	ATMT	○	SI	×	Zhou et al. 2022
Apple	ATMT	○	SI	○	Chen et al. 2022a
Wheat	ATMT	○	SI	○	Qiu et al. 2022
Peanut	ATMT	×	SI	×	Zhai et al. 2022
Tobacco	VVMT	×	SI	×	Ellison et al. 2020
Tomato	VVMT	○	SI	×	Liu et al. 2023a, b
Sweet potato	VVMT	×	TT	×	Zhang et al. 2024
Tobacco	VVMT	-	TT	×	Cody et al. 2017
Tobacco	NMT	-	TT	×	Torney et al. 2007
Maize	NMT	-	SI	×	Wang et al. 2022b
*Eruca sativa*	NMT	×	TT	×	Kwak et al. 2019
Poplar	NMT	-	TT	×	Hoengenaert et al. 2023
Wheat, Tobacco, Arugula, and Cotton	NMT	-	TT	×	Demirer et al. 2019
Tomato	PBT	○	SI	×	Tanwar et al. 2024
Chinese cabbage	PBT	○	SI	×	Liu et al. 2024b
Arabidopsis	PMT	-	TT	×	Yoo et al. 2007
*Kalanchoe blossfeldiana*	PMT	○	SI	×	Castelblanque et al. 2010

*ATMT* *Agrobacterium tumefaciens*-mediated transformation, *ARMT* *Agrobacterium rhizogenes*-mediated transformation, *VVMT* Virus vector mediated transformation, *NMT* Nanoparticle mediated transformation, *PBT* Particle bombardment transformation, *PMT* PEG mediated transformation, *SI* Stable inheritance, *TT* Transient transformation

**Table 2:** Comparison of different regeneration systems of the same plant transformation

Species	Tissue culture regeneration system				Non-tissue culture regeneration system				Practicality evaluation
	Transformation efficiency (%)	Transformation duration (week)	Delivery system	References	Transformation efficiency (%)	Transformation duration (week)	Delivery system	References	
Sweet potato	1 ~ 5	24 ~ 40	Agrobacterium mediated	Choi et al. 2007; Yu et al. 2007; Yang et al. 2011	10 ~ 40	3 ~ 15	Agrobacterium mediated	Cao et al. 2022; Mei et al. 2024; Zhang et al. 2024	Simple operation, high efficiency, and short duration
*Taraxacum kok-saghyz*	10 ~ 79	> 20	Agrobacterium mediated	Dai et al. 2021	40 ~ 50	10 ~ 12	Agrobacterium mediated	Cao et al. 2022	Simple operation and short duration
*Coronilla varia*	45	> 13	-	Mariotti and Arcioni. 1983	3	-	Agrobacterium mediated	Cao et al. 2022	Simple operation but low efficiency
*Ailanthus altissima*	23	28	-	Ichihashi and Kako. 1977	39	-	Agrobacterium mediated	Cao et al. 2022	Simple operation and high efficiency
*Aralia elata*	80	19	-	-	2	-	Agrobacterium mediated	Cao et al. 2022	Low efficiency
*Taraxacum mongolicum*	< 20	18	Agrobacterium mediated	Lee et al. 2004	16 ~ 25	2 ~ 3	Agrobacterium mediated	Cao et al. 2024	Simple operation and short duration
*Rehmannia glutinosa*	-	-	-	-	12 ~ 20	4 ~ 8	Agrobacterium mediated	Cao et al. 2024	First implementation
Wheat	45 ~ 62	8 ~ 12	Agrobacterium mediated	Yu et al. 2024	12.9 ~ 100	9 ~ 12	Agrobacterium mediated	Li et al. 2021	Simple operation
*Sansevieria trifasciata*	20 ~ 90	21 ~ 26	-	Kaur et al. 2021	3.9 ~ 7.8	12 ~ 16	Agrobacterium mediated	Lu et al. 2024	Short duration but low efficiency
Peanut	50	15	Agrobacterium mediated	Zhou et al. 2022	50	7	Agrobacterium mediated	Zhai et al. 2022	Short duration
Potato	1	-	Agrobacterium mediated	Vinterhalter et al, 2009	1 0~ 40	2 ~ 3	Agrobacterium mediated	Mei et al. 2024	Simple operation and high efficiency
*Kalanche blossfeldiana*	6	37	PEG mediated	Castelblanque et al. 2010	74	8	Agrobacterium mediated	Lu et al. 2024	Simple operation, high efficiency, and short duration
*Eruca sativa*	-	-	-	-	35 ~ 88	3.5	Nanoparticle mediated	Kwak et al. 2019	First implementation
Tobacco	45 ~ 95	-	Agrobacterium mediated	Shoyeb et al. 2020	65 ~ 80	2.5	Virus vector mediated	Ellison et al. 2020	First implementation

## Data Availability

Not applicable.
